# Role of PKCε in the epithelial-mesenchymal transition induced by FGFR2 isoform switch

**DOI:** 10.1186/s12964-020-00582-1

**Published:** 2020-05-19

**Authors:** Danilo Ranieri, Monica Nanni, Flavia Persechino, Maria Rosaria Torrisi, Francesca Belleudi

**Affiliations:** 1 Department of Clinical and Molecular Medicine, Sapienza University of Rome, Laboratory Affiliated to Istituto Pasteur Italia – Fondazione Cenci Bolognetti, Viale Regina Elena 324, 00161 Rome, Italy; 2 S. Andrea University Hospital, Rome, Italy

**Keywords:** FGFR2c, PKCε, Epithelial-mesenchymal transition, Keratinocytes

## Abstract

**Background:**

The epithelial isoform of the fibroblast growth factor receptor 2 (FGFR2b) controls the entire program of keratinocyte differentiation via the sequential involvement of protein kinase C (PKC) δ and PKCα. In contrast, the FGFR2 isoform switch and the aberrant expression of the mesenchymal FGFR2c isoform leads to impairment of differentiation, epithelial-mesenchymal transition (EMT) and tumorigenic features. Aim of our present study was to contribute in clarifying the complex network of signaling pathways involved in the FGFR2c-mediated oncogenic outcomes focusing on PKCε, which appears to be involved in the induction of EMT and tumorigenesis in several epithelial contexts.

**Methods:**

Biochemical and molecular analysis, as well as in vitro invasion assays, combined with the use of specific small interfering RNA (siRNA), were performed in human keratinocytes stably expressing FGFR2c or FGFR2b isoforms.

**Results:**

Our results showed that aberrant expression and signaling of FGFR2c, but not those of FGFR2b, in human keratinocytes induced a strong phosphorylation/activation of PKCε. The use of siRNA approach showed that PKCε is the hub signaling downstream FGFR2c responsible for the modulation of EMT markers and for the induction of the EMT-related transcription factors STAT3, Snail1 and FRA1, as well as for the acquisition of the invasive behavior. Moreover, experiments of depletion of ESRP1, responsible for FGFR2 splicing in epithelial cells, indicated that the activation of PKCε is the key molecular event triggered by FGFR2 isoform switch and underlying EMT induction.

**Conclusions:**

Overall, our results point to the identification of the downstream PKC isoform responsible for the FGFR signaling deregulation occurring in epithelial tissues from the physiological oncosoppressive to the pathological oncogenic profile.

Video Abstract

**Graphical abstract:**

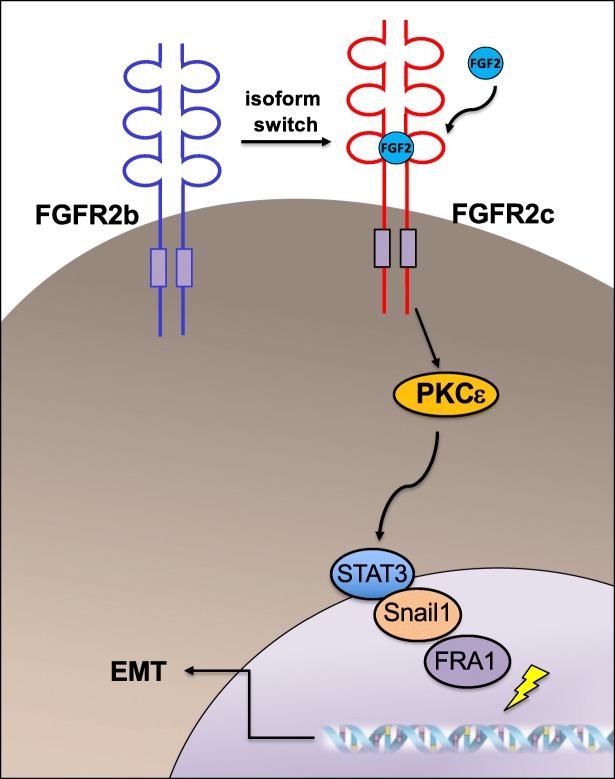

## Background

It is known that most of the human genes undergo alternative splicing and many studies have suggested that the isoform switch represent a crucial event in cancer [1]. In this regard, several studies have demonstrated that the switching from the epithelial isoforms of fibroblast growth factor receptors (FGFR1-3b) to the mesenchymal FGFR1-3c isoforms is frequently involved in epithelial-mesenchymal transition (EMT) and cancer progression [2–4]. In the last years we focused our attention on the biological functions of the epithelial FGFR2b isoform, demonstrating that this receptor controls the entire program of keratinocyte differentiation [5–7] through the sequential involvement of PKCδ and PKCα signaling [7]. In contrast, we found that the FGFR2 isoform switch and the aberrant expression of the mesenchymal FGFR2c isoform in the same epidermal context induced changes in FGFR ligand specificity, leading to impairment of differentiation [8], EMT and early tumorigenic features [9, 10]. In addition, we have also recently shown the negative impact of the out-of-context expression of FGFR2c on autophagy [11], consistent with a possible role of this receptor in the modulation of the proposed EMT/autophagy negative loop during carcinogenesis. However, although the multiple functional impacts of the aberrant expression of FGFR2c begin to be progressively described, the specific downstream signaling network underlying these oncogenic outcomes remain still to be identified. With the aim to contribute in dissecting this network, here we paid attention on PKCs. In particular, we focused on PKCε, which has been found overexpressed in several carcinomas, including squamous cell carcinomas (SCCs) [12, 13]. Moreover, PKCε shows the greatest oncogenic potential among PKC family members [13] and it has been proposed to play a relevant role in EMT induction [14, 15]: in fact, PKCε overexpression alone is sufficient to dramatically increase growth rate and motility in human keratinocytes (HKs) [16], as well as to induce EMT-related phenotype in non-tumorigenic mammary epithelial cells [14, 15], strongly encouraging us to go deeper inside on its possible function as key molecular player in the context of aberrant FGFR2c expression and signaling.

## Methods

### Cells and treatments

The human keratinocyte cell line HaCaT, stably expressing FGFR2c (pBp-FGFR2c), overexpressing FGFR2b (pBp-FGFR2b) or the empty vector (pBp) were cultured in Dulbecco’s modified eagle’s medium (DMEM), supplemented with 10% fetal bovine serum (FBS) plus antibiotics.

For RNA interference and PKCε or ESRP1 silencing, cells were transfected with PKCε small interfering RNA (PKCε siRNA) (Santa Cruz Biotechnology, Inc., Santa Cruz, CA, USA; sc36251), ESRP1 siRNA (Santa Cruz Biotechnology, SC77526), or an unrelated siRNA as a control, using Lipofectamine 2000 transfection reagent (Life Technologies, Carlsbad, CA, USA; 11,668–019) according to the manufacturer’s protocol.

For growth factors stimulation, cells were left untreated or incubated with FGF7 (Upstate Biotechnology, Lake Placid, NY, 01–118) or with FGF2 (PeproTech, London, BFGF 100–188) 25 ng/mL for 24 h at 37 °C. To induce activation and signaling of FGFR2 isoforms, cells were serum starved and incubated with FGF7 or FGF2 100 ng/mL for 10 min at 37 °C. For inhibition of FGFR2b and FGFR2c tyrosine kinase activity, cells were pre-incubated with a specific FGFR tyrosine kinase inhibitor, SU5402 25 μM (Calbiochem, Nottingham, UK; 572,630) for 1 h before treatments with growth factors (GFs).

### Invasion assay

Migration assay was performed using 24-well transwell migration Boyden chambers (8 μm pore size; Costar, Cambridge, MA, USA) precoated with matrigel (dilution 1:2 in DMEM; BD Biosciences, Bedford, MA, USA). 5X 10^4^ cells were seeded in each filter and serum starved for 4 h at 37 °C. To induce chemotaxis: FGF2 25 ng/ml was added to the lower chamber. After 48 h, cells on the upper side of membranes were removed, while cells migrated on the bottom side were fixed in methanol and stained with toluidine blue. Quantitative analysis was assessed counting for each sample the migrated cells in 10 microscopic fields (objective used: 20X) from three independent experiments. Results have been expressed as mean values ± SD. *p* values were calculated using Student’s t test and significance level has been defined as *p* > 0.05.

### Western blot analysis

Cells were lysed in a buffer containing 50 mM HEPES, pH 7.5, 150 mM NaCl, 1% glycerol, 1% Triton X-100, 1.5 mM MgCl2, 5 mM EGTA, supplemented with protease inhibitors (10 g/mL aprotinin, 1 mM phenylmethylsulfonyl fluoride [PMSF], 10 μg/mL leupeptin) and phosphatase inhibitors (1 mM sodium orthovanadate, 20 mM sodium pyrophosphate, 0.5 M NaF). A range of 20 to 50 μg of total protein was resolved under reducing conditions by 8 or 12% SDS-PAGE and transferred to reinforced nitrocellulose (BA-S 83; Schleicher & Schuell, Keene, NH, USA; BA-S83). The membranes were blocked with 5% nonfat dry milk (Bio-Rad Laboratories, Hercules, CA, USA, 170–6404) in PBS 0.1% Tween 20 (Bio-Rad, 170–6531) and incubated with anti-p-PKCδ (Ser 645, Abcam, Cambridge, UK, ab108972), anti-E-cadherin (NCH-38, Dako, Carpinteria, CA, USA), anti-β4-integrin (7, Santa Cruz Biotechnology, sc-135,950), anti N-cadherin (Sigma-Aldrich, Saint Louis, MO, USA, C3865), monoclonal antibodies or with anti Bek (C17, Santa Cruz Biotechnology), p-PKCε (Ser729, Abcam, Cambridge, UK, ab63387), anti ESRP1 (Sigma-Aldrich, HPA023719), polyclonal antibodies, followed by enhanced chemiluminescence (ECL) detection (Thermo Scientific, Rockford, IL, USA; 34,580). The membranes were rehydrated by washing in PBS/Tween-20, stripped with 100 mM mercaptoethanol and 2% SDS for 30 min at 55 °C and probed again with, anti-PKCε (Abcam, ab124806), anti-PKCδ (Santa Cruz Biotechnology) polyclonal antibodies, or with anti-actin (Sigma-Aldrich, A5441) monoclonal antibody to estimate the protein equal loading. Densitometric analysis was performed using Quantity One Program (Bio-Rad). The resulting values from three different experiments were normalized and expressed as fold increase respect to the control value. Values from a representative of three independent experiments were reported in each figure. The student’s *t* test was performed and significance levels have been defined as *p* < 0.05.

### Primers

Oligonucleotide primers were purchased from Invitrogen (Carlsbad, CA, USA). The following primers were used: for the PKCε target gene: 5′- GGTGAAGCCCCTAAAGACAATG-3′ (sense), 5′-GACCTGATGGACCCTGCG-3′ (antisense); for the PKCδ target gene: 5′-CGCATCGCCTTCAACTCCTA-3′ (sense), 5′-AGTGTTTTCCCACGCTCTGT-3′ (antisense); for the E-cadherin target gene: 5′-TGGAGGAATTCTTGCTTTGC-3′ (sense), 5′-CGCTCTCCTCCGAAGAAAC-3′ (antisense); for the β4-integrin target gene: 5′-GGGAAAAAGCAAGACCACACC-3′ (sense), 5′-CCCTCTGTTCCACCTGCTTC-3′ (antisense); for the vimentin target gene: 5′-AAATGGCTCGTCACCTTCGT-3′ (sense), 5′- AGAAATCCTGCTCTCCTCGC-3′ (antisense); for the Snail1 target gene: 5′-GCTGCAGGACTCTAATCCAGA-3′ (sense), 5′-ATCTCCGGAGGTGGGATG-3′ (antisense); for the STAT3 target gene: 5′-CAGAGATGTGGGAATGGGGG-3′ (sense), 5′- TGGCAAGGAGTGGGTCTCTA-3′ (antisense); for the FRA1 target gene: 5′- GCAGGCGGAGACTGACAAA-3′ (sense), 5′-GATGGGTCGGTGGGCTTC-3′, for ESRP1 target gene: 5′-GGCTCGGATGAGAAGGAGTT-3′ (sense), 5′-GCACTTCGTGCAACTGTCC-3′ (antisense); for FGFR2b target gene: 5′-CGTGGAAAAGAACGGCAGTAAATA-3′ (sense), 5′-GAACTATTTATCCCCGAGTGCTTG-3′ (antisense); for FGFR2c target gene: 5′- TGAGGACGCTGGGGAATATACG-3′ (sense), 5′-TAGTCTGGGGAAGCTGTAATCTCCT 3′ (antisense); for the 18S rRNA housekeeping gene: 5′-CGAGCCGCCTGGATACC-3′ (sense) and 5′-CATGGCCTCAGTTCCGAAAA-3′ (antisense). For each primer pair, we performed no-template control and no-reverse-transcriptase control (reverse transcription [RT]-negative) assays, which produced negligible signals.

### RNA extraction and cDNA synthesis

RNA was extracted using the TRIzol method (Invitrogen, 15,596,018) according to the manufacturer’s instructions and eluted with 0.1% diethylpyrocarbonate (DEPC)-treated water. Each sample was treated with DNase I (Invitrogen, 18,068–015). The total RNA concentration was quantitated by spectrophotometry; 1 μg of total RNA was used for reverse transcription using the iScriptTM cDNA synthesis kit (Bio-Rad, 170–8891) according to the manufacturer’s instructions.

### PCR amplification and real-time quantitation

Real-time RT-PCR was performed using the iCycler real-time detection system (iQ5 Bio-Rad) with optimized PCR conditions. The reactions were carried out in a 96-well plate using iQ SYBR green supermix (Bio-Rad, 1,708,882), adding forward and reverse primers for each gene and 1 μl of diluted template cDNA to a final reaction mixture volume of 15 μl. All assays included a negative control and were replicated three times. Real-time quantitation was performed with the help of the iCycler IQ optical system software, version 3.0a (Bio-Rad), according to the manufacturer’s manual. Results are reported as mean values ± SE from three different experiments in triplicate. The student’s *t* test was performed, with significance levels defined as *P* values < 0.05.

## Results

### PKCε signaling is responsible for FGFR2c-mediated modulation of EMT-related markers

**Fig. 1:**
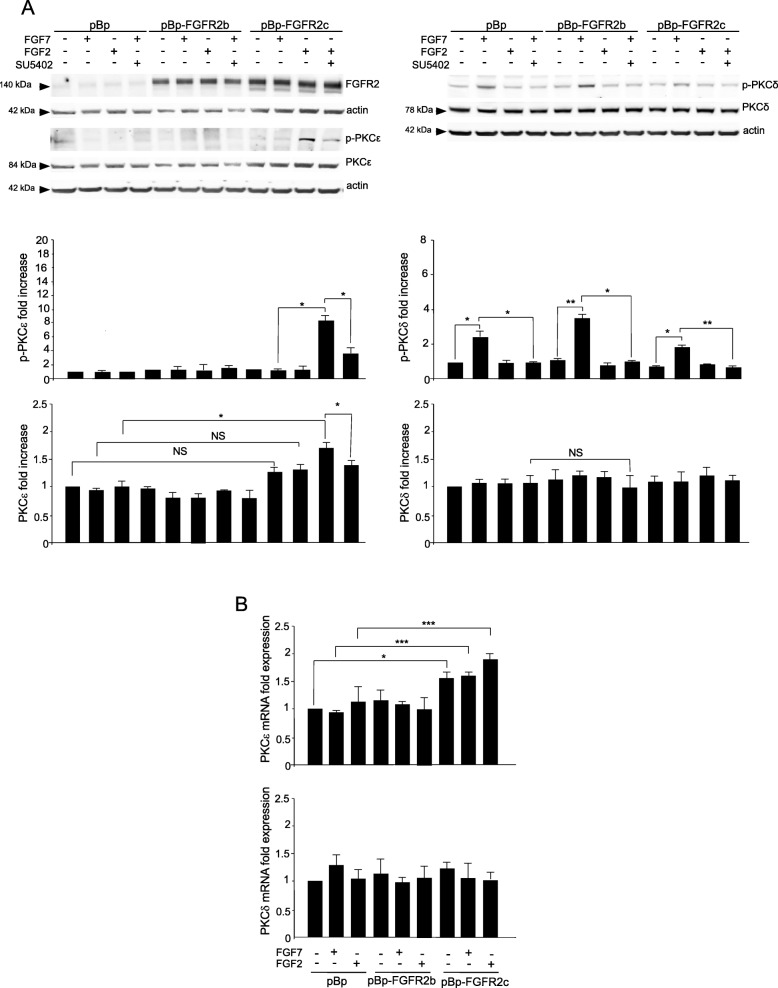
FGFR2c aberrant expression and signaling induce PKCε activation. HaCaT clones stably transduced with pBp-FGFR2c or with pBp-FGFR2b or empty pBp vector, used as controls, were left untreated or stimulated with FGF7 or with FGF2 in presence or absence of the FGFR tyrosine kinase inhibitor SU5402 as described in Material and Methods. **a** Western blot analysis shows that the 140 KDa band corresponding to the molecular weight of FGFR2 is mainly evident in cells transduced with FGFR2b or FGFR2c isoforms. An appreciable phosphorylation of PKCε at the autophosphorylation site Ser 729 is observed only in pBp-FGFR2c clones upon FGF2 stimulation and this effect is abolished by SU5402. A moderate up-regulation of PKCε protein level is detectable only in pBp-FGFR2c clones, particularly in response to FGF2. A moderate phosphorylation of PKCδ at the autophosphorylation site Serine 645, is visible in all clones in response to FGF7 stimulation, while no evident modulation of PKCδ protein level is observed. Equal loading was assessed for p-PKCε with the anti-PKCε antibody, for p-PKCδ with the anti-PKCδ antibody, for PKCε and PKCδ with the anti-actin antibody. For the densitometric analysis, the values from 3 independent experiments were normalized, expressed as fold increase and reported in graph as mean values ± standard deviation (SD). Student *t* test was performed and significance levels have been defined as *p* < 0.05: *p < 0.05, ** *p* < 0.01. **b** Real time RT-PCR analysis shows a positive modulation of PKCε expression at mRNA transcript level only in pBp-FGFR2c clones, particularly in response to FGF2. No evident modulation of PKCδ mRNA expression is observed. Results are expressed as mean value ± standard error (SE). Student’s *t* test was performed and significance levels were defined as *p* < 0.05: *p < 0.05, *** *p* < 0.001

In order to verify whether PKCε could be responsible for the multiple oncogenic outcomes of aberrant FGFR2c expression, we first assayed the ability of this receptor to impact on PKCε activity. To this aim, we took advantage of the human keratinocyte HaCaT clones stably transduced with pBp-FGFR2c retroviral constructs or with pBp-FGFR2b or empty pBp vector, used as controls [10]. Cells were left untreated or stimulated with FGF7, the specific ligand of FGFR2b, or with FGF2, which does not bind to FGFR2b, but is able to activate other FGFRs including FGFR2c. To assess the involvement of PKCε, we verified its phosphorylation in Ser 729 in the C-terminal hydrophobic motif, which depends on the internal catalytic activity of the kinase and is a widely recognized indicator of PKCε activation [17, 18]. Western blot analysis showed that an appreciable phosphorylation of PKCε at the autophosphorylation site Ser 729 was visible only in HaCaT pBp-FGFR2c clones upon FGF2 stimulation (Fig. 1a) and this effect was abolished by the presence of the specific FGFR tyrosine kinase inhibitor SU5402 (Fig. 1a). Thus, PKCε activation could be, in our cell model, specifically ascribed to the FGFR2c expression and signaling. In addition, the moderate increase of PKCε at both protein (Fig. 1a) and mRNA transcript levels (Fig. 1b), detectable only in pBp-FGFR2c clones, particularly in response to FGF2, suggested that FGFR2c activation also induced an appreciable up-regulation of this protein. The phosphorylation of PKCδ at the autophosphorylation site Serine 645, which belongs to the characteristic phosphorylation pattern of PKCδ activation [19] was observed in all clones only in response to FGF7 (Fig. 1a), was in agreement with our recent data proposing a key role of this PKC family member in the early steps of FGF7-mediated keratinocyte differentiation [7]. No evident modulation of both PKCδ protein (Fig. 1a) and mRNA (Fig. 1b) was detected in all clones, as expected [7].

**Fig. 2:**
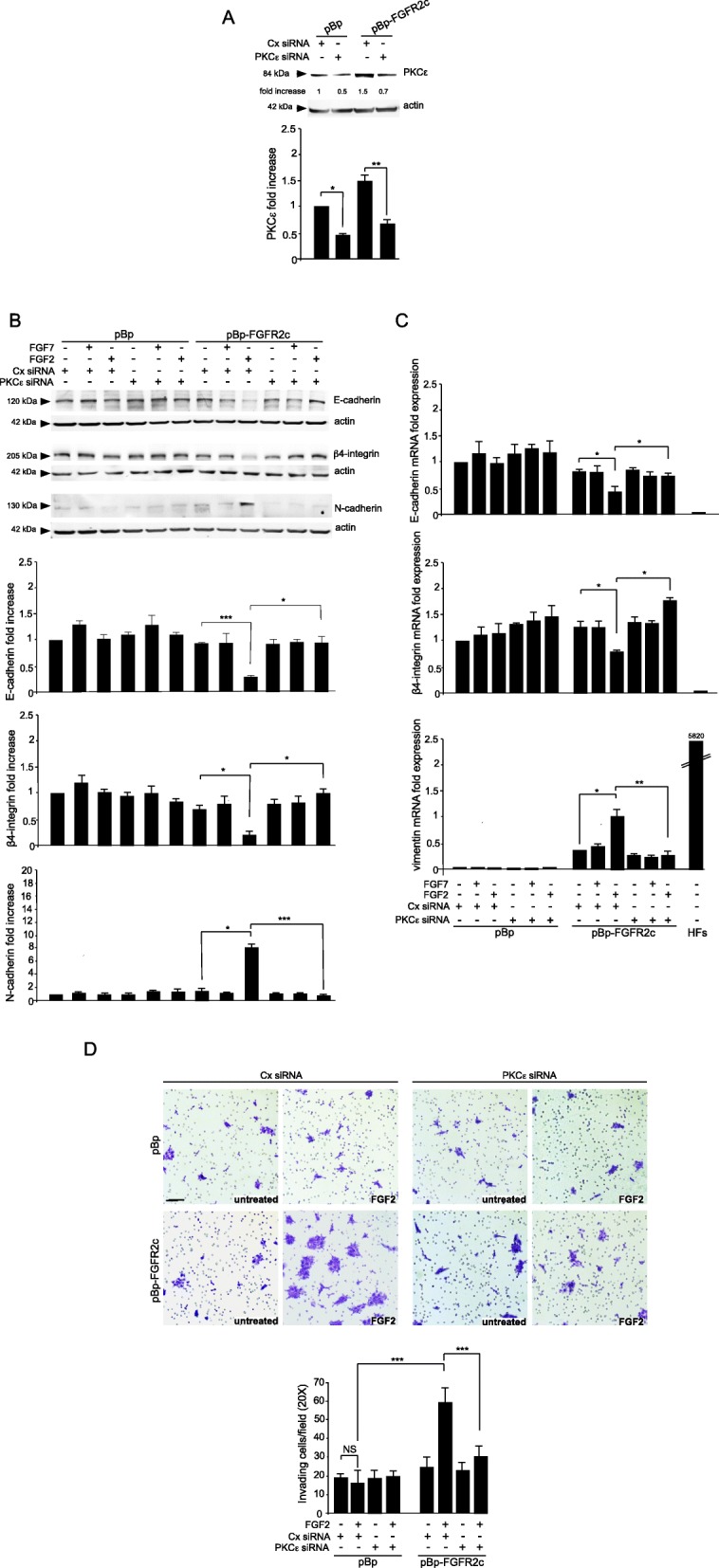
PKCε signaling is responsible for FGFR2c-mediated modulation of EMT markers. HaCaT pBp and HaCaT pBp-FGFR2c clones were transfected with PKCε small interfering RNA (siRNA) or with an unrelated siRNA (Cx siRNA) as control, and then left untreated or stimulated with FGFR2 ligands as above. **a** Western blot shows that in untreated cells PKCε siRNA transfection induces an efficient depletion of PKCε. Equal loading was assessed with the anti-actin antibody. The densitometric analysis and Student *t* test were performed as reported in Fig. 1: *p < 0.05, ** *p* < 0.01. **b** Western blot analysis shows that the decrease of the epithelial markers E-cadherin and β4-integrin and the appearance of the mesenchymal marker N-cadherin, observed only in pBp-FGFR2c clones upon FGF2 stimulation, is reversed by PKCε silencing. Equal loading was assessed with the anti-actin antibody. The densitometric analysis and Student *t* test were performed as reported above: *p < 0.05, *** *p* < 0.001. **c** Real Time RT-PCR analysis shows that, in pBp-FGFR2c cultures, PKCε depletion is sufficient to counteract the FGF2-induced mRNA transcript modulation of E-cadherin, β4-integrin and the mesenchymal marker vimentin. Human Fibroblasts (HFs) are used as a positive control for mesenchymal marker expression. Results are expressed as mean value ± SE. Student’s *t* test was performed and significance levels were defined as *p* < 0.05: *p < 0.05, ** *p* < 0.01. **d** HaCaT clones were seeded on matrigel pre-coated transwell Boyden chamber filters. Cells were then serum starved and FGF2 was added in the bottom chamber to stimulate cell chemotaxis. An high number of invasive cells is induced by FGF2 stimulation only in pBp-FGFR2c control siRNA clones and appears strongly reduced in the corresponding PKCε siRNA cultures. Quantitative analysis was assessed as reported in materials and methods. Results are expressed as mean values ± standard deviation (SD). Student’s t test was performed as reported in materials and methods and significance level has been defined as *p* < 0.05: ***p < 0.001. Bar: 50 μm

To assess the PKCε contribution in FGFR2c-mediated modulation of EMT marker expression, we performed its depletion via small-interfering RNA approaches. HaCaT pBp-FGFR2c and HaCaT pBp clones were transfected with PKCε siRNA or with an unrelated siRNA as control (Cx siRNA) and the efficiency of protein depletion was first verified by Western blot analysis (Fig. 2a). Transfected cells were left untreated or stimulated with FGFR2 ligands as above. Western blot analysis showed that both the decrease of the epithelial markers E-cadherin and β4-integrin and the appearance of the mesenchymal marker N-cadherin, evident only in pBp-FGFR2c clones by FGF2 stimulation, were reversed by PKCε silencing (Fig. 2b). These findings were also validated by Real Time RT-PCR analysis, showing that, in pBp-FGFR2c cultures, PKCε depletion was sufficient to counteract the FGF2-induced mRNA transcript modulation of E-cadherin, β4-integrin and the mesenchymal marker vimentin (Fig. 2c).

Based on our previous findings on the role of FGFR2c in conferring invasiveness in the well-established non-invasive HaCaT cells [8, 10] we assessed the impact of PKCε depletion on this acquired ability. To this aim we analyzed the capacity of HaCaT pBp-FGFR2c and HaCaT pBp cells to migrate through transwell Boyden chambers pre-coated with a thin layer of matrigel, a gel composed of reconstituted basement membrane elements resembling the basement membrane in vivo. Upon seeding, cells were left untreated or stimulated with FGF2 for 48 h. Since the stimulation with FGF7 did not appear to impact on EMT-related marker expression in both pBp and FGFR2c clones (Fig. 2b, c), this treatment was not performed. The results showed that the significant increase of invading cells, observed only in FGFR2c cultures in response to FGF2 (Fig. 2d), was clearly impaired by PKCε silencing (Fig. 2d). Thus, the expression and possibly activation of PKCε appears to significantly contribute to the appearance of early tumorigenic features.

### PKCε signaling acts via the induction of the EMT-related transcription factors

**Fig. 3:**
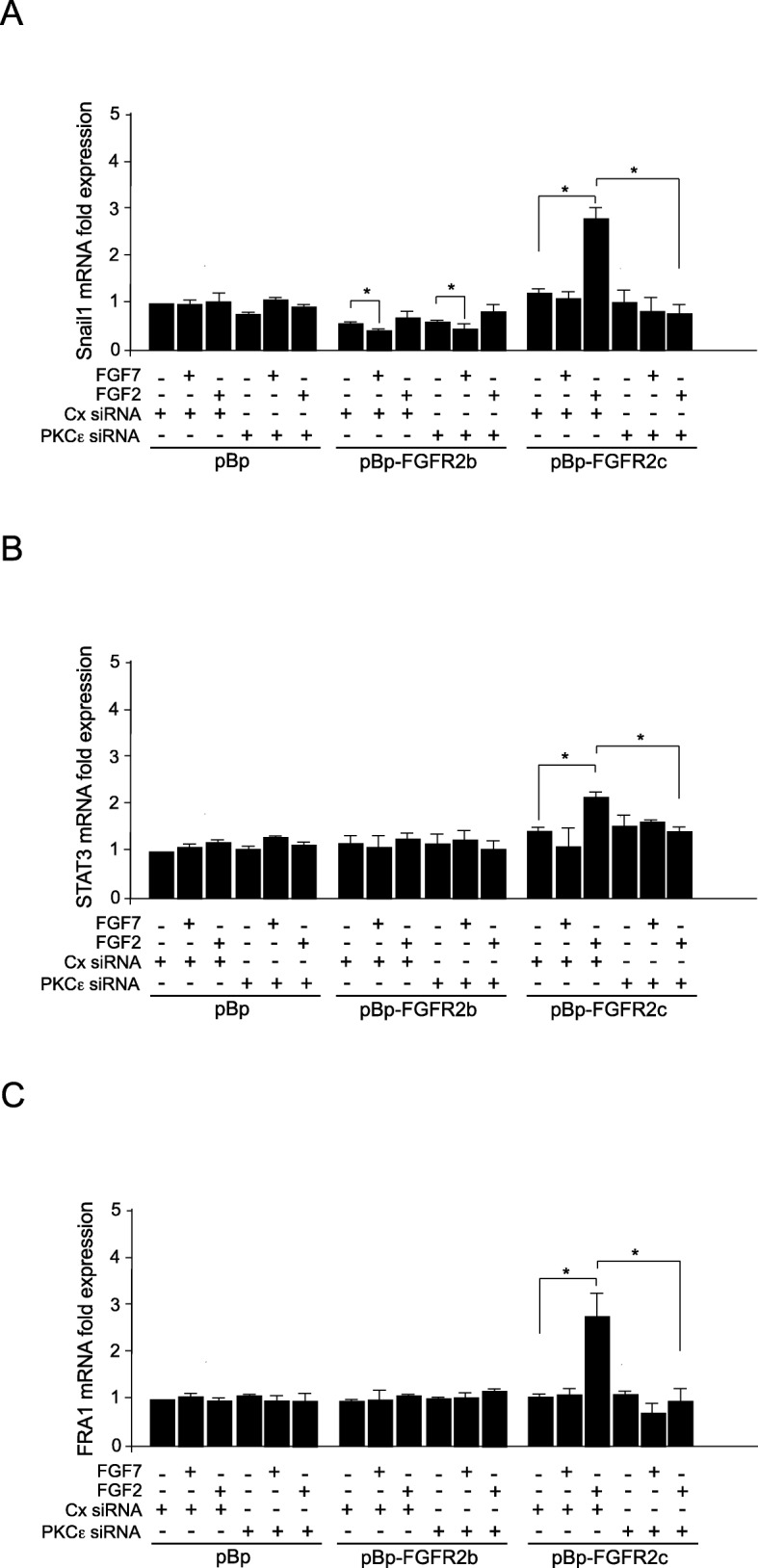
PKCε signaling is responsible for FGFR2c-mediated induction of the EMT-related transcription factors. Clones were transfected with PKCε siRNA or with Cx siRNA and left untreated or stimulated with FGFR2 ligands as above. **a** Real-time RT-PCR shows that the increase of the master transcription factor for EMT Snail1, evident only in pBp-FGFR2c clones following FGF2 stimulation, is abolished by PKCε silencing. On the contrary, in pBp-FGFR2b clones Snail1 transcript expression is slightly repressed, particularly in response to FGF7, while PKCε silencing does not affect this trend. **b** A significant induction of STAT3 mRNA transcript level is observed in pBp-FGFR2c clones stimulated with FGF2 and this effect is abolished by PKCε silencing. No modulations of RNA transcripts are observed in control clones in response to FGF7. **c** Real-time RT-PCR shows that FRA1 mRNA transcript levels are significantly induced by FGF2 stimulation only in FGFR2c-expressing culture. This effect appears significantly inhibited by PKCε depletion. No effects are observed in control clones in response to FGF7. Results are expressed as mean value ± SE. Student’s *t* test was performed and significance levels were defined as *p* < 0.05. *p < 0.05

We have previously demonstrated that the aberrant signaling of FGFR2c in the epithelial context induces expression of Snail1 [10], which is the master transcription factor for EMT [20, 21] and mainly associated with the EMT onset [22]. Therefore, to assess whether the depletion of PKCε protein could be able to block the initiation of FGFR2c-induced EMT, expression of Snail1 mRNA was investigated in all HaCaT clones transfected with PKCε siRNA or with control siRNA and stimulated with FGFR2 ligands as above. Real-time RT-PCR showed that the increase of Snail1 mRNA expression, evident only in pBp-FGFR2c clones following FGF2 stimulation, was abolished by PKCε silencing (Fig. 3a). As expected [10] and in agreement with its proposed role as tumor suppressor, the overexpression of FGFR2b isoform slightly repressed Snail1 transcript expression, particularly in response to its ligand FGF7 (Fig. 3a), while PKCε silencing did not affect this trend (Fig. 3a). Thus, the selective shut-off of PKCε signaling is ineffective on FGFR2b-dependent modulation of Snail1, but it appears to be sufficient to inhibit its FGFR2c-mediated induction.

It has been previously described that, during EMT, Snail1 can be induced by the transcription factor STAT3. Interestingly, STAT3, which is up-regulated in several human carcinomas, including head and neck squamous cell carcinoma (HNSCC), breast, ovary, prostate, and lung cancer [23–27], is specifically activated by PKCε [12, 28]. Since STAT3 is also induced and activated by several FGFRs, including FGFR2 [29], we wondered whether, in the context of human keratinocytes aberrantly expressing FGFR2c, this transcription factor could act downstream PKCε and possibly upstream Snail1. Real time RT-PCR showed a significant induction of STAT3 in pBp-FGFR2c clones stimulated by FGF2 (Fig. 3b), which was abolished by PKCε silencing (Fig. 3b), while no modulations of RNA transcripts were detected in control clones in response to FGF7, even when FGFR2b was overexpressed (Fig. 3b). Thus, STAT3 appears to be an effector exclusive for the FGFR2c isoform, whose activation take place downstream PKCε.

Because EMT is also triggered by Snail-dependent induction of FRA1 [30], another transcription factor activated by FGFRs, including FGFR2 [31], we wondered if FRA1 would be also induced by PKCε during FGFR2c-driven EMT. Real-time RT-PCR showed that, similarly to what observed for Snail1 and STAT3, FRA1 mRNA transcripts were significantly induced by FGF2 stimulation only in FGFR2c-expressing culture (Fig. 3c) and this effect appeared significantly inhibited by PKCε depletion (Fig. 3c). No modulating effects were observed in control clones in response to FGF7 suggesting that, similarly to STAT3 and Snail1, also FRA1 is an exclusive effector of FGFR2c (Fig. 3c).

### The activation of PKCε is the key molecular event triggered by FGFR2 isoform switch and underlying EMT induction

**Fig. 4:**
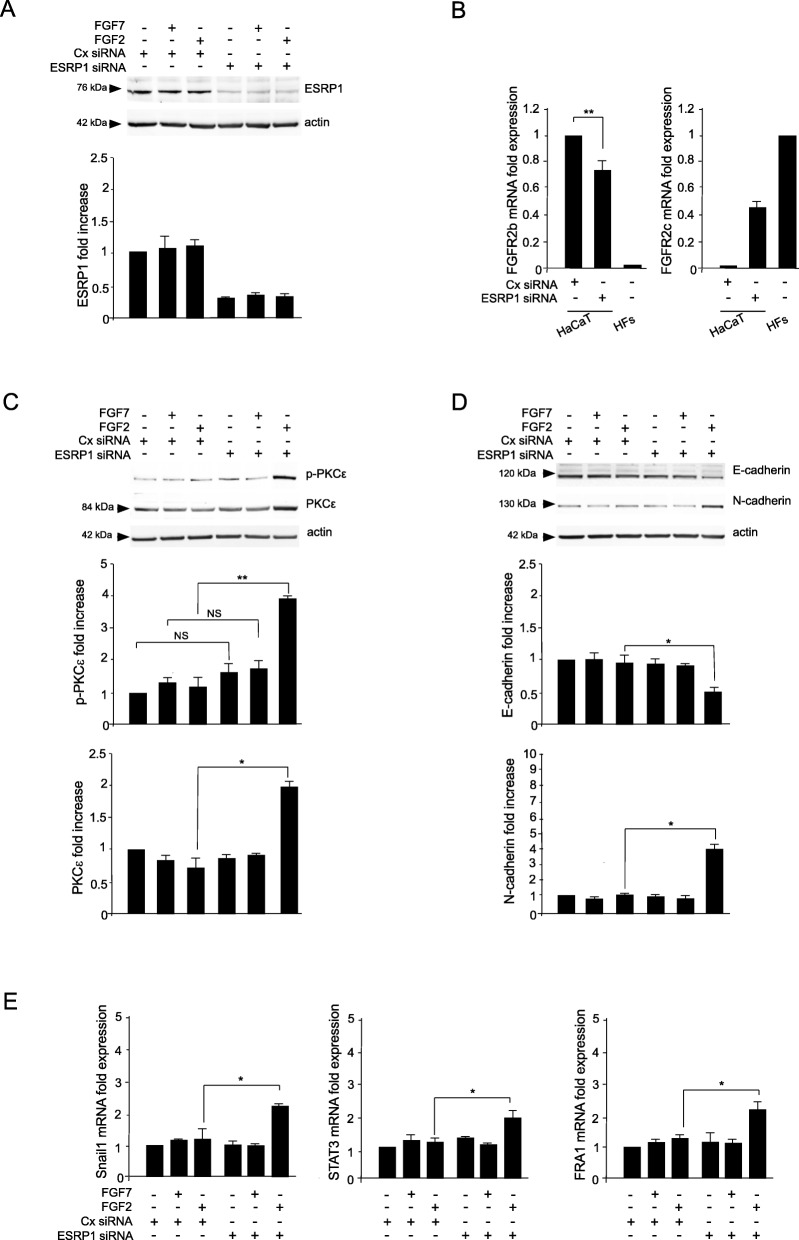
FGFR2 isoform switch triggers PKCε activation, as well as induction of EMT. HaCaT cells were transfected with siRNA for ESRP1 or with an unrelated siRNA (Cx siRNA), as control, and then left untreated or stimulated with FGF7 or FGF2, as above. **a** Western blot analysis shows that ESRP1 siRNA induces an efficient depletion of ESRP1. Equal loading was assessed with the anti-actin antibody. The densitometric analysis was performed as reported above. **b** Real-time RT-PCR shows that ESRP1 silencing leads to the decrease of FGFR2b expression and to the appearance of FGFR2c mRNA transcript levels. HFs are used as a positive control for FGFR2c expression. Results are expressed as mean value ± SE. Student’s *t* test was performed and significance levels were defined as *p* < 0.05. **p < 0.01. **c**, **d** Biochemical approaches show that ESRP1 depletion is sufficient to make HaCaT cells responsive to FGF2 in terms of PKCε activation/phosphorylation and EMT induction, as demonstrated by E-cadherin repression and N-cadherin appearance. Equal loading was assessed with the anti-actin antibody. The densitometric analysis and Student *t* test were performed as reported above: *p < 0.05, ** *p* < 0.01. **e** Real-time RT-PCR shows that ESRP1 depletion, upon FGF2 stimulation, induces the up-regulation of the three EMT-related transcription factors Snail1, STAT3 and FRA1. Results are expressed as mean value ± SE. Student’s *t* test was performed and significance levels were defined as *p* < 0.05. *p < 0.05

**Fig. 5:**
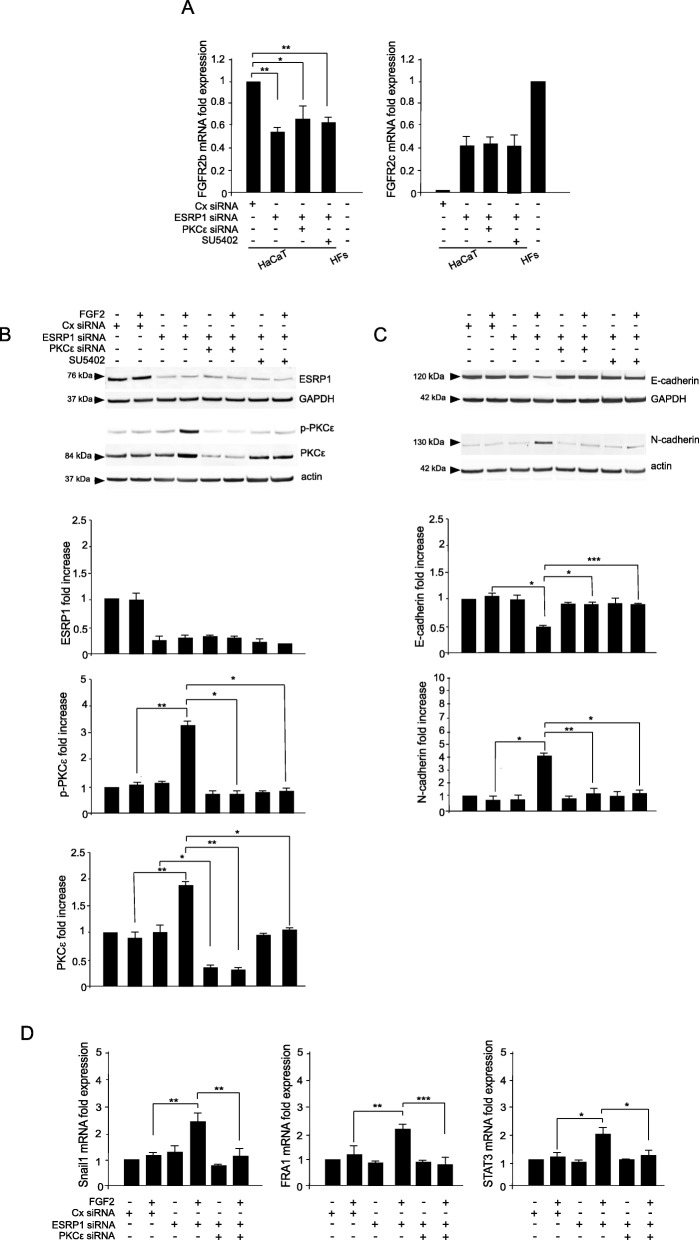
PKCε depletion, as well as the inhibition of FGFR2c kinase activity counteract FGFR2 isoform switch-induced EMT. HaCaT cells were transfected with siRNA for ESRP1 or co-trasfected with with siRNA for ESRP1 and siRNA for PKCε. The transfection with unrelated siRNA (Cx siRNA) was used as control. Cells were then left untreated or stimulated with FGF2 in presence or absence of SU5402 as above. The densitometric analysis was performed as reported above. **a** Real-time RT-PCR shows that the decrease of FGFR2b expression and to the appearance of FGFR2c mRNA transcripts induced ESRP1 silencing is not affected by PKCε depletion or by the presence of SU5402. Results are expressed as mean value ± SE. Student’s *t* test was performed and significance levels were defined as *p* < 0.05. *p < 0.05, **p < 0.01. **b**, **c** Biochemical approaches show that ESRP1 and PKCε siRNA induces an efficient depletion of ESRP1 and PKCε protein levels (**b**). PKCε activation/phosphorylation (**b**), as well as E-cadherin downregulation and N-cadherin appearance (**c**), induced by ESRP1 depletion, are efficiently inhibited by both the depletion of PKCε or the treatment with SU5402. The densitometric analysis and Student *t* test were performed as reported above: *p < 0.05, ** *p* < 0.01, *** *p* < 0.001. **d** Real-time RT-PCR shows that the depletion of PKCε is sufficient to block the induction of the EMT-related transcription factors, evident in ESRP1-depleted cells in response to FGF2. Results are expressed as mean value ± SE. Student’s *t* test was performed as above. *p < 0.05, ** *p* < 0.01, *** *p* < 0.001

In order to assess if the switching from the epithelial FGFR2b versus the mesenchymal FGFR2c isoform could represent the specific event responsible for PKCε activation and consequent EMT induction in human keratinocytes, we performed the depletion of the epithelial splicing regulatory protein1 (ESRP1) which is responsible for the FGFR2 splicing and consequent expression of FGFR2b in epithelial context [9, 32]. HaCaT cells were transfected with ESRP1 siRNA or with an unrelated siRNA (Cx siRNA), as control, and then stimulated with FGF7 or FGF2, as reported above. The efficiency of ESRP1 depletion was verified by biochemical approaches (Fig. 4a). Then, real time RT-PCR was performed to assess the decrease of FGFR2b expression (Fig. 4b, left panel) and the appearance of FGFR2c (Fig. 4b, right panel) following ESRP1 depletion, confirming that the correct splicing of the FGFR2 gene, occurring in epithelial context, has been impaired. Then, focusing our attention on PKCε signaling, Western blot analysis showed that ESRP1 depletion and consequent FGFR2 isoform switch was sufficient to make HaCaT cells responsive to FGF2 in term of PKCε activation/phosphorylation (Fig. 4c). This event was accompanied by EMT induction, as demonstrated by E-cadherin repression and N-cadherin appearance (Fig. 4d), as well as by the up-regulation of the three EMT-related transcription factors STAT3, Snail1 and FRA1 (Fig. 4e). The effects observed after ESRP1 depletion were comparable to those observed in HaCaT clones expressing FGFR2c (see Figs. 1, 2 and 3). Interestingly, all the above effects triggered by FGF2 after ESRP1 depletion were significantly counteracted by both the co-depletion of PKCε and/or the treatment with the FGFR2 inhibitor SU5402 (Fig. 5b-d), confirming that FGFR2c activation and PKCε signaling take place downstream FGFR2 isoform switch and are required for EMT induction. In contrast, neither PKCε silencing or SU5402 treatment appear to be able to interfere with FGFR2 isoform switch induced by ESRP1 depletion (Fig. 5a).

## Discussion

To clarify how the diversification of the complex network of receptor tyrosine kinase (RTK) signaling is regulated is presently considered a crucial topic, since the impairment of this regulation is increasingly described in several diseases, including cancer. In particular for FGFRs, the isoform switching is one of the proposed ways for signaling deregulation involved in both epithelial-mesenchymal transition (EMT) and cancer progression [2–4]. In this regard, previous works from our group have demonstrated that FGFR2 isoform switch and the consequent aberrant expression of the mesenchymal FGFR2c isoform in human keratinocytes is responsible for impairment of differentiation [8], EMT induction and appearance of early tumorigenic features [9, 10]. However, clear knowledge of the molecular players of the oncogenic signaling established by the aberrant expression of the mesenchymal FGFR2c isoform in the epithelial context is still lacking.

The findings reported in the present work appears to indicate that PKCε activation could be a key molecular signaling event occurring as a consequence of FGFR2c expression. In particular, the experiments of depletion of ESRP1, involved in FGFR2 splicing in epithelial cells, indicated that the activation of PKCε is the key molecular event triggered by FGFR2 isoform switch and underlying EMT induction in human keratinocytes. Our findings are consistent with several evidences indicating that expression of FGFR2c correlates with EMT induction and with the acquisition of a more aggressive, invasive phenotype in different epithelial tumor cell lines [33]. Interestingly, in some of these cell lines, such as prostate cancer cells, a crucial role has been also in parallel proposed for PKCε in the induction of EMT and cell invasion [15].

In this scenario, PKCε would act upstream an oncogenic signaling network, leading to the induction of EMT-related transcription factors such as STAT3, Snail1 and FRA1, which are probably induced in cascade and cooperate with each other in triggering and sustaining the EMT program.

Interestingly, the different specificity in PKC isoform activation displayed by FGFR2b and FGFR2c in our cellular model is consistent with the recent advanced hypothesis that the alternative splicing could represent the emerging mechanism to diversify FGFR signaling [34]. In fact, splicing events might alter the FGFR2 interactions with a huge number of other membrane proteins, such as other RTKs or G-protein-coupled receptors (GPCRs), impacting on the downstream scaffold protein formation and consequently on the signaling transduced by these multiprotein complexes at the plasma membrane [33]. In light of these assumptions, it is reasonable suppose that the formation of these complexes could affect also the relocalization of FGFRs and of their signaling substrates to specific subcellular environments, containing the appropriate downstream targets: this could be essential to determine the output of a specific signaling cascade [34].

Among the different RTK signaling players, PKCs are a class of finely regulated serine-threonine kinases that are essential for the control of higher-level signal organization as well as spatial distribution of the signals [13, 35]. In fact, it has been proposed that PKCs can be recruited to membrane protein scaffolds, where they may control the behavior of protein complexes influencing their assembly state, but also their subcellular localization and their ability to recruit different downstream effectors [35]. This is particularly true for PKCε for which many cell-type- and context-specific signaling functions have been demonstrated to regulate several cellular processes not only via the phosphorylation of multiple and alternative downstream substrates, but also through their relocalization to different intracellular sites [36].

## Conclusions

Taken all together our results, which point to the identification of downstream PKC isoforms exclusive for each of the FGFR2 isoforms, such as PKCδ for FGFR2b and PKCε for FGFR2c, represent the first step to advance our understanding of the molecular bases of FGFR signaling deregulation occurring in epithelial tissues from the physiological oncosoppressive to the pathological oncogenic profile.

## Data Availability

The dataset used and/or analyzed during the current study are available from the corresponding author on reasonable request.

## Abbreviations

DAPI: 4′,6-diamidino-2-phenylindole
EMT: Epithelial-mesenchymal transition
ESRP: Epithelial Splicing Regulatory Protein
FGF2: Fibroblast growth factor 2 (basic)
FGF7/KGF: Fibroblast growth factor 7
FGFR: Fibroblast growth factor receptor
GPCRs: G-protein-coupled receptors
HaCaT: Human adult skin keratinocytes propagated under low calcium
HNSCC: Head and neck squamous cell carcinoma
HFs: Human fibroblasts
HKs: Human keratinocytes
PKC: Protein kinase C
RTK: Receptor tyrosine kinase
SCC: Squamous cell carcinomas
SD: Standard deviation
SDS-PAGE: Sodium dodecyl sulphate-polyacrylamide gel electrophoresis
SE: Standard error
siRNA: Small interfering RNA

## References

[1] Wang BD, Lee NH (2018). Aberrant RNA splicing in cancer and drug resistance. Cancers..

[2] Oltean S, Sorg BS, Albrecht T, Bonano VI, Brazas RM, Dewhirst MW (2006). Alternative inclusion of fibroblast growth factor receptor 2 exon IIIc in dunning prostate tumors reveals unexpected epithelial mesenchymal plasticity. Proc Natl Acad Sci U S A.

[3] Shirakihara T, Horiguchi K, Miyazawa K, Ehata S, Shibata T, Morita I (2011). TGF-β regulates isoform switching of FGF receptors and epithelial-mesenchymal transition. EMBO J.

[4] Zhao Q, Caballero OL, Davis ID, Jonasch E, Tamboli P, Yung WK (2013). Tumor-specific isoform switch of the fibroblast growth factor receptor 2 underlies the mesenchymal and malignant phenotypes of clear cell renal cell carcinomas. Clin Cancer Res.

[5] Belleudi F, Purpura V, Torrisi MR (2011). The receptor tyrosine kinase FGFR2b/KGFR controls early differentiation of human keratinocytes. PLoS One.

[6] Purpura V, Belleudi F, Caputo S, Torrisi MR (2013). HPV16 E5 and KGFR/ FGFR2b interplay in differentiating epithelial cells. Oncotarget.

[7] Rosato B, Ranieri D, Nanni M, Torrisi MR, Belleudi F (2018). Role of FGFR2b expression and signaling in keratinocyte differentiation: sequential involvement of PKCδ and PKCα. Cell Death Dis.

[8] Ranieri D, Rosato B, Nanni M, Belleudi F, Torrisi MR (2018). Expression of the FGFR2c mesenchymal splicing variant in human keratinocytes inhibits differentiation and promotes invasion. Mol Carcinog.

[9] Ranieri D, Belleudi F, Magenta A, Torrisi MR (2015). HPV16 E5 expression induces switching from FGFR2b to FGFR2c and epithelial-mesenchymal transition. Int J Cancer.

[10] Ranieri D, Rosato B, Nanni M, Magenta M, Belleudi F, Torrisi MR (2016). Expression of the FGFR2 mesenchymal splicing variant in epithelial cells drives epithelial-mesenchymal transition. Oncotarget.

[11] Nanni, Ranieri, Persechino, Torrisi, Belleudi (2019). The Aberrant Expression of the Mesenchymal Variant of FGFR2 in the Epithelial Context Inhibits Autophagy. Cells.

[12] Gorin MA, Pan Q (2009). Protein kinase C epsilon: an oncogene and emerging tumor biomarker. Mol Cancer.

[13] Isakov N (2018). Protein kinase C (PKC) isoforms in cancer, tumor promotion and tumor suppression. Semin Cancer Biol.

[14] Jain K, Basu A (2014). Protein kinase C-ε promotes EMT in breast cancer. Breast Cancer (Auckl).

[15] Jain K, Basu A (2014). The multifunctional protein kinase C-ε in cancer development and progression. Cancers (Basel).

[16] Papp G, Czifra E, Bodó J, Lázár I, Kovács M, Aleksza I (2004). Opposite roles of protein kinase C isoforms in proliferation, differentiation, apoptosis, and tumorigenicity of human HaCaT keratinocytes. Cell Mol Life Sci.

[17] Lau E, Kluger H, Varsano T, Lee KY, Scheffler I, Rimm DL (2012). PKCε promotes oncogenic functions of ATF2 in the nucleus while blocking its apoptotic function at mitochondria. Cell.

[18] Karavana VN, Gakiopoulou H, Lianos EA (2014). Expression of Ser729 phosphorylated PKCepsilon in experimental crescentic glomerulonephritis: an immunohistochemical study. Eur J Histochem.

[19] Zhang L, Keane MP, Zhu LX, Sharma S, Rozengurt E, Strieter RM (2004). Interleukin-7 and transforming growth factor-beta play counter-regulatory roles in protein kinase C-delta-dependent control of fibroblast collagen synthesis in pulmonary fibrosis. J Biol Chem.

[20] Thiery JP, Acloque H, Huang RY, Nieto MA (2009). Epithelial- mesenchymal transitions in development and disease. Cell.

[21] Lamouille S, Xu J, Derynck R (2014). Molecular mechanisms of epithelial-mesenchymal transition. Nat Rev Mol Cell Biol.

[22] Tran DD, Corsa CA, Biswas H, Aft RL, Longmore GD (2011). Temporal and spatial cooperation of Snail1 and Twist1 during epithelial–Mesenchymal transition predicts for human breast cancer recurrence. Mol Cancer Res.

[23] Chan KS, Sano S, Kiguchi K, Anders J, Komazawa N, Takeda J (2004). Disruption of Stat3 reveals a critical role in both the initiation and the promotion stages of epithelial carcinogenesis. J Clin Invest.

[24] Alvarez JV, Febbo PG, Ramaswamy S, Loda M, Richardson A, Frank DA (2005). Identification of a genetic signature of activated signal transducer and activator of transcription 3 in human tumors. Cancer Res.

[25] Rivat C, Rodrigues S, Bruyneel E, Piétu G, Robert A, Redeuilh G (2005). Implication of STAT3 signaling in human colonic cancer cells during intestinal trefoil factor 3 (TFF3)—and vascular endothelial growth factor-mediated cellular invasion and tumor growth. Cancer Res.

[26] Aziz MH, Manoharan HT, Church DR, Dreckschmidt NE, Zhong W, Oberley TD (2007). Protein kinase Cepsilon interacts with signal transducers and activators of transcription 3 (Stat3), phosphorylates Stat3Ser727, and regulates its constitutive activation in prostate cancer. Cancer Res.

[27] Kobielak A, Fuchs E (2006). Links between a-catenin, NF- nB, and squamous cell carcinoma in skin. Proc Natl Acad Sci U S A.

[28] Aziz MH, Manoharan HT, Verma AK (2007). Protein kinase C epsilon, which sensitizes skin to sun’s UV radiation-induced cutaneous damage and development of squamous cell carcinomas, associates with Stat3. Cancer Res.

[29] Dudka AA, Sweet SM, Heath JK (2010). Signal transducers and activators of transcription-3 binding to the fibroblast growth factor receptor is activated by receptor amplification. Cancer Res.

[30] Tam WL, Lu H, Buikhuisen J, Soh BS, Lim E, Reinhardt F (2013). Protein kinase C α is a central signaling node and therapeutic target for breast cancer stem cells. Cancer Cell.

[31] Mathur A, Ware C, Davis L, Gazdar A, Pan BS, Lutterbach B (2014). FGFR2 is amplified in the NCI-H716 colorectal cancer cell line and is required for growth and survival. PLoS One.

[32] Warzecha CC, Sato TK, Nabet B, Hogenesch JB, Carstens RP (2009). ESRP1 and ESRP2 are epithelial cell-type-specific regulators of FGFR2 splicing. Mol Cell.

[33] Ishiwata T (2018). Role of fibroblast growth factor receptor-2 splicing in normal and cancer cells. Front Biosci.

[34] Latko Marta, Czyrek Aleksandra, Porębska Natalia, Kucińska Marika, Otlewski Jacek, Zakrzewska Małgorzata, Opaliński Łukasz (2019). Cross-Talk between Fibroblast Growth Factor Receptors and Other Cell Surface Proteins. Cells.

[35] Rosse C (2010). PKC and the control of localized signal dynamics. Nat Rev Mol Cell Biol.

[36] Newton PM, Messing RO (2010). The substrates and binding partners of protein kinase Cepsilon. Biochem J.

